# Reduction of Lung Metastases by Na[*trans*-RuCl_4_(DMSO)Im] is not Coupled With the Induction of Chemical Xenogenization

**DOI:** 10.1155/MBD.1996.67

**Published:** 1996

**Authors:** G. Sava, G. Salerno, A. Bergamo, M. Cocchietto, R. Gagliardi, E. Alessio, G. Mestroni

**Affiliations:** 1 Fondazione Cellerio Institute of Biological Researsh via A. Fleming 22-31 Trieste I-34127 Italy; 2 Departement of chemical sciences via L. Giorgieri 7-9 University of Trieste Italy

## Abstract

The effects of the treatment of tumor cells of MCa mammary carcinoma and TLX5 lymphoma with the ruthenium complex Na[*trans*-RuCl_4_ (DMSO)lm] for several transplant generations were studied on tumor growth and metastases formation. On TLX5 lymphoma cells, treatment was performed *in vitro* prior to *in vivo* inoculation of tumor cells in intact or immunesuppressed mice. Either considering tumor take and growth or its capacity to invade the brain of the inoculated hosts, Na[*trans*-RuCl_4_(DMSO)lm] did not induce any significant modification. Conversely, in mice with MCa mammary carcinoma, the *in vivo* treatment of tumor cells in immunesuppressed hosts caused a progressive increase of DNA activity and, starting from the 4th transplant generation, a significantly increased susceptibility of lung metastasis formation to a further treatment in intact mice. These data seem to suggest that Na[*trans*-RuCl_4_(DMSO)Im] does not induce chemical xenogenization of tumor cells nor its repeated treatment induces resistance in tumor cells. Conversely, it appears that Na[*trans*-RuCl_4_(DMSO)lm] may select a tumor cell population which maintains its capacity to metastasise to the lung but with enhanced sensitivity to the antimetastatic properties of this compound.


### REDUCTION OF LUNG METASTASES BY Na[*trans*-$RuCl_4$(DMSO)Im] IS NOT COUPLED WITH THE INDUCTION OF CHEMICAL XENOGENIZATION

G. Sava[1^*], G. Salerno[1], A. Bergamo[1], M. Cocchietto[1], R. Gagliardi[1], E. Alessio[2], and G. Mestroni[2]

[1] Fondazione Callerio, Institute of Biological Research via A. Fleming 22-31, I-34127 Trieste

[2] Department of Chemical Sciences via L. Giorgieri 7-9, University of Trieste, Italy

**ABSTRACT**

The effects of the treatment of tumor cells of MCa mammary carcinoma and TLX5 lymphoma with the ruthenium complex Na[*trans*-$RuCl_4$(DMSO)Im] for several transplant generations were studied on tumor growth and metastases formation. On TLX5 lymphoma cells, treatment was performed *in vitro* prior to *in vivo* inoculation of tumor cells in intact or immunesuppressed mice. Either considering tumor take and growth or its capacity to invade the brain of the inoculated hosts, Na[*trans*-$RuCl_4$(DMSO)Im] did not induce any significant modification. Conversely, in mice with MCa mammary carcinoma, the *in vivo* treatment of tumor cells in immunesuppressed hosts caused a progressive increase of DNA activity and, starting from the 4th transplant generation, a significantly increased susceptibility of lung metastasis formation to a further treatment in intact mice. These data seem to suggest that Na[*trans*-$RuCl_4$(DMSO)Im] does not induce chemical xenogenization of tumor cells nor its repeated treatment induces resistance in tumor cells. Conversely, it appears that Na[*trans*-$RuCl_4$(DMSO)Im] may select a tumor cell population which maintains its capacity to metastasise to the lung but with enhanced sensitivity to the antimetastatic properties of this compound.

**INTRODUCTION**

The effects of a new generation ruthenium(III) complex, Na[*trans*-$RuCl_4$(DMSO)Im], are different from those of cisplatin in that, unlike cisplatin that is equally active on primary tumor growth and lung colonies, Na[*trans*-$RuCl_4$(DMSO)Im] is markedly effective only on spontaneous metastases (1-3). The selectivity of Na[*trans*-$RuCl_4$(DMSO)Im] on lung metastases is marked also on advanced metastases and accounts for a significant prolongation of host's survival time, particularly in the experiments in which drug treatment is associated with surgical removal of primary tumor. This effect is not associated with any residual effect on primary tumor cells after treatment discontinuation whereas it tends to reduce the metastatic ability of the same tumor (3).

Either by means of *vivo-vivo* bioassays or by microscopical examination it appears that the growth of lung tumors is markedly reduced whereas the growth of the i.m. primary tumor is much less affected and histologically not detectable. These effects account for the prolongation of the survival time and for the cure rate observed and highlight the pharmacological properties of this compound for the control of solid tumor metastases, an effect that was shown to be similarly exerted also on advanced tumor metastases (4,5).

Metastases represent the greatest obstacle to cures after surgery and/or radiotherapy in that they often show a low chemosensitivity to the available anticancer drugs (6). The lack of success derives from the fact that tumor metastases are always treated with drugs that have been specifically developed by studying their activity in reducing primary tumor growth rather than by examining their efficacy on the more selective metastatic population (7). The aim of the present investigation was that of examining the cumulative effects of repeated treatments for several transplant generations on primary tumor growth and lung metastasis formation using the MCa mammary carcinoma of CBA mouse. Tumor treatment will be performed *in vivo* either in mice immunesuppressed by DTIC or in intact hosts. Parallelly, *in vitro* treatments of TLX5 lymphoma cells for up to 13 transplant generations will be performed to ascertain the possible occurrence of chemical xenogenizing effects, similar to those caused by triazeno derivatives and nitrosoguanidine derivatives (8-10). This study will therefore

^ Permanent address: Department of Biomedical Sciences, University of Trieste, via L. Giorgieri 7-9, 34127, Trieste Italy. E-mail: CALLERIO@univ.trieste.it.

focus the attention on the possible intervention of antigenic modifications on tumor cells after treatment and on the susceptibility of treated tumor cells to further treatments in terms of modification of tumor growth and metastasis formation.

### MATERIALS AND METHODS

Compounds and treatments. Na[*trans*-$RuCl_4$(DMSO)Im] was prepared according to standard procedures (9,10). The compound was dissolved in 0.9% NaCl and was administered i.p. to mice in volumes of 0.1 ml/10 g body weight. The dose of Na[*trans*-$RuCl_4$(DMSO)Im] (100 mg/Kg/day), at the treated schedule used, is well tolerated by the treated animals and does not cause appreciable reduction of body weight gain *vs* untreated controls at the end of treatment.
MCa mammary carcinoma. The line of MCa mammary carcinoma of CBA mouse was obtained from the Rudjer Boskovich Institute, Zagreb, Croatia (11), and was maintained by biweekly passages of $10^6$ viable tumor cells into the calf of the left hind leg of CBA inbred female mice of 20ñ2 g obtained from a locally established breeding colony. Tumor propagation for experimental purposes was similarly carried out using female mice 6-8 weeks old. Tumor cell suspensions were prepared from primary tumors of donors similarly inoculated two weeks before. In short, 2.5 g of freshly removed tumor was minced with scissors, finely dispersed in 20 ml Dulbecco's phosphate saline calcium and magnesium free (PBS) and filtered through a double layer of sterile gauze; after centrifuging at 250xg per 10 min, pelletts were resuspended in an equal volume of PBS and cell viability was checked by the trypan blue exclusion test: only cell suspensions with at least 55-60% viable cells were used.
Primary tumor and lung metastasis evaluation. Primary tumors were measured by caliper and their weight estimated by the following formula: $(\pi/6)a^2b$ [Equation 1], where a and b are two perpendicular axes (a<b) and assume tumor density equal to 1. The evaluation of the number and weight of lung metastases, spontaneously formed from the s.c. tumor implants, was performed after the killing of the animals by cervical dislocation. The number of lung metastases on the surface of the freshly removed lungs was counted by means of a low-power stereo microscope equipped with a calibrated grid. The weight of the metastatic tumor per mouse was calculated by determining the volume of each metastatic nodule by the formula of primary tumors reported above [Equation 1].
Histological analysis. Pieces of primary tumor were collected immediately after killing of the animals and fixed in formalin. For light transmission observations, slices were stained with hematoxylin-eosin or with Cajal-Callego mounted in Canada Balsam, and were observed in double blind with a Leitz-Orthoplan microscopy.
TLX5 lymphoma. The TLX5 lymphoma line, originally obtained from the Chester Beatthy Research Institute, London, England, was maintained by weekly passages of $10^5$ cells/mouse i.p. into CBA mice. For the experimental purposes, the tumor was collected from the peritoneal cavity of donor mice inoculated one week before, and washed twice with PBS.
*In vitro-in vivo* bioassays. Tumor cell suspensions of TLX5 lymphoma ($10^6$ cells/ml) were kept *in vitro* at 37°C for 60 min under shaking in tissue culture tubes with 1.9 ml of PBS containing antibiotics (100 U/ml penicillin and 100 mg/ml streptomycin) to which were added 100 ml PBS containing the test compound for a final volume of 1.5 ml. At the end of the incubation, aliquots of 0.1 ml were injected i.p. into intact syngenic CBA mice of which survival time was recorded.
Brain metastases. The determination of the occurrence of leukemic brain involvement was performed by means of a *vivo-vivo* bioassay. Briefly, whole brains of mice transplanted with TLX5 lymphoma cells one week before, were aseptically removed after killing of the animal by cervical dislocation. Brains were subsequently transplanted s.c. in the flank of intact syngenic CBA mice by means of a sterile syringe with a 19x21 needle. The survival time of the transplanted mice gave an indirect measure of the amount of TLX5 lymphoma cells present in the transplanted brains (12,13).
Cytofluorimetric analysis. Propidium iodide staining was performed according to the procedure described by Krishan (14). Orange acridine staining was performed according to the methods of Darzynkiewicz (15).
Statistical analysis. All data were subjected to statistical analysis by means of the computerized Student-Newmann-Keuls test or the t-test for grouped data. Statistical differences were accepted at the cut-off threshold of at least $p<0.05$.

Animal studies. Animal studies were carried out according to the guidelines actually in force in Italy and according to the Guide for the Care and Use of Laboratory Animals. DHHS Publ. No (NIH) 86-23. Bethesda, Md: NIH, 1985.

### RESULTS

Effects on primary tumor and metastasis formation. The effects of the administration of 100 mg/kg/day Na[*trans*-$RuCl_4$(DMSO)Im] on days 1,5,9,13 after i.m. implantation of $2x10^6$ MCa mammary carcinoma cells on day 0 in CBA mice immunesuppressed by 240 mg/kg Dacarbazine on day -2, are evaluated by measuring the growth of primary tumor and of spontaneous lung metastases in two further groups of CBA animals (untreated or treated with the same dose and treatment schedule of Na[*trans*-$RuCl_4$(DMSO)Im]) [generation 1]) to which the treated tumor cells were transplanted following removal on day 14. This experiment was continued for up to 8 transplant generations (Figure 1, Panels B and C).

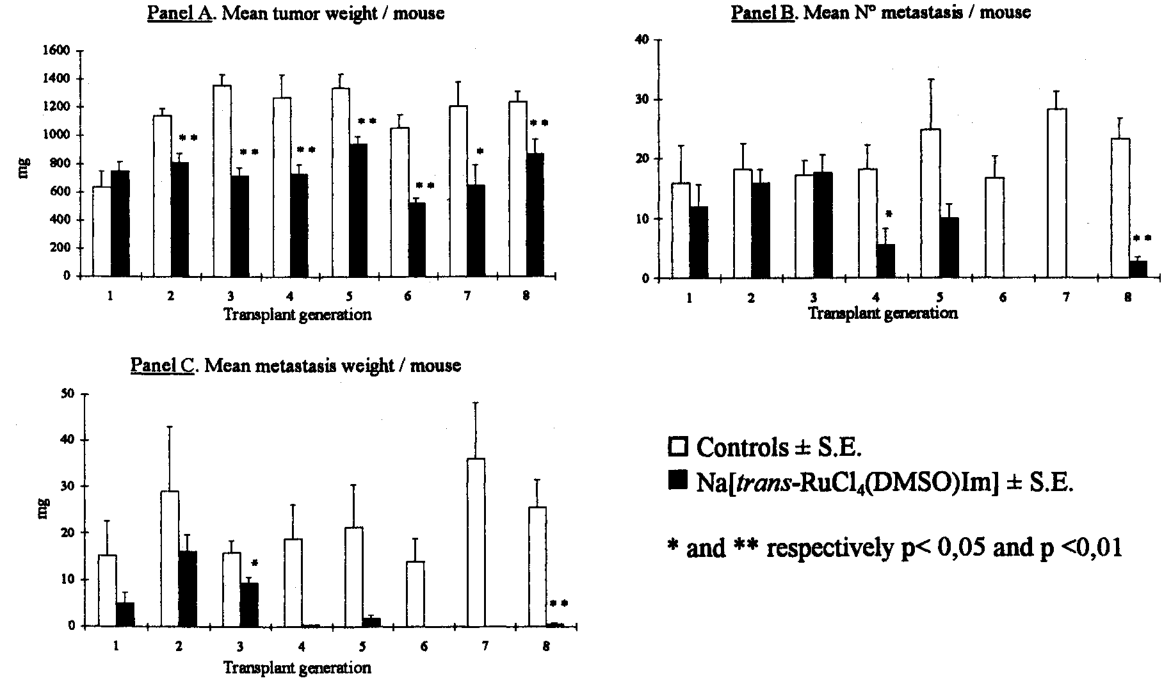

**Figure 1.** Effects of *in vivo* treatment of MCa mammary carcinoma with Na[*trans*-$RuCl_4$(DMSO)Im] for 8 transplant generations on primary tumor growth and on lung metastasis formation. Groups of 5 CBA mice, implanted i.m. with $10^6$ MCa mammary carcinoma cells on day 0 and immunosuppressed with 240 mg/kg DTIC i.p. on day -2, were given Na[*trans*-$RuCl_4$(DMSO)Im] (100 mg/kg/day on days 1,5,9,13). On day 14, primary tumors were removed, pooled and transplanted into two groups (A and B) of 8 intact CBA syngenic hosts each (transplant generation 1) and into a group of 5 CBA mice immunosuppressed as above (C). Groups B and C were further treated with the same dose and treatment schedule of Na[*trans*-$RuCl_4$(DMSO)Im]; group C was further processed as the initial treated group giving transplant generation 2 and so on. Primary tumor growth was measured on day 14 and lung metastasis formation was evaluated on day 25 after tumor transplantation.

The analysis of data obtained from all 8 transplant generations shows no significant modification of the response of primary tumor to Na[*trans*-$RuCl_4$(DMSO)Im], whereas lung metastasis formation was markedly affected starting from generation 4 onwards. A detail of the curve of primary tumor growth and of the reduction of spontaneous lung metastases is given in Figure 2, Panel B. The growth of MCa mammary carcinoma in untreated mice is regular at all transplant generations and is not statistically different of that of the intact untreated MCa mammary carcinoma, parental line in both intact and immunosuppressed mice. An example of the curves of tumor growth is given in Figure 2, Panel A.

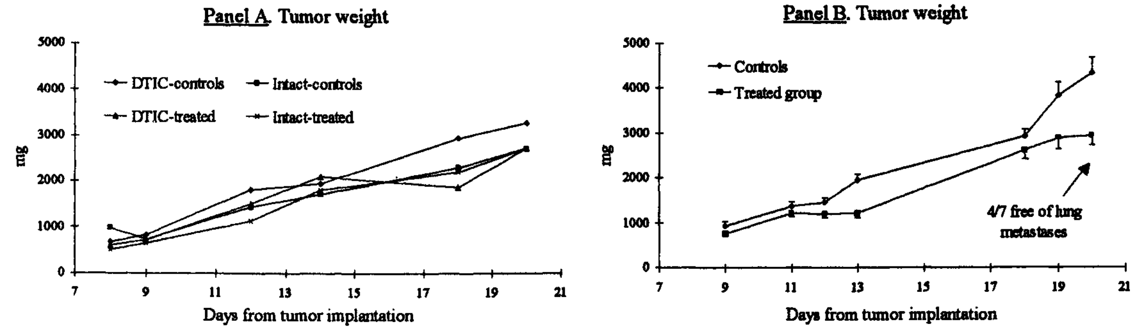

**Figure 2.** Comparison of the growth of the primary tumor of MCa mammary carcinoma untreated or treated with Na[*trans*-$RuCl_4$(DMSO)Im].Groups of 5 CBA mice intact or immunosuppressed with DTIC, implanted with $2x10^6$ MCa mammary carcinoma cells obtained from the primary tumor of immunosuppressed mice untreated or treated with Na[*trans*-$RuCl_4$(DMSO)Im] (100 mg/kg/day on days 1,5,9,13) for 6 transplant generations (Panel A) or for 4 transplant generations (Panel B), were examined for primary tumor growth.

Effects on MCa polyploidy. The measurement of MCa mammary carcinoma polyploidy was performed 24 hr after last drug administration in intact mice transplanted with MCa mammary carcinoma treated in immunesuppressed hosts. Treatment by Na[*trans*-$RuCl_4$(DMSO)Im] of the MCa mammary carcinoma in intact hosts line of generations 1-8 does not significantly modify the proportion of 2n, 4n and 8n population compared with untreated controls (Figure 3); the proportion of cells is equivalent to that of the parental tumor line.

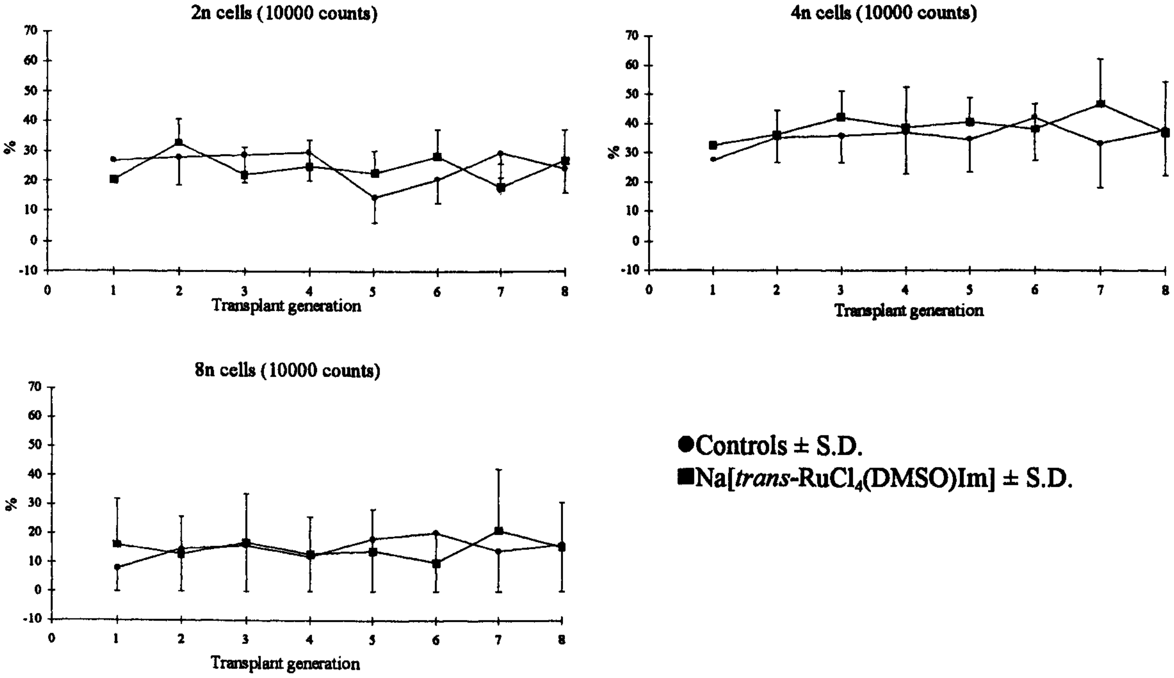

**Figure 3.** Effects of *in vivo* treatment of MCa mammary carcinoma with Na[*trans*-$RuCl_4$(DMSO)Im] for 8 transplant generations on tumor cell ploidy. 3 mice of groups A and B of transplant generations of Figure 1 were killed on day 14 and primary tumors removed, pooled and processed for the preparation of a tumor cell suspension stained with propidium iodide and read with the cytofluorimeter.

Effects on DNA-RNA activity in MCa carcinoma cells. Data reported in Figure 4 show that treatment of MCa mammary carcinoma by Na[*trans*-$RuCl_4$(DMSO)Im] in immunesuppressed hosts causes a progressive increase of DNA and only a minor effect on of RNA activity (Figure 4, Panel A). This effect is maintained in intact hosts trasplanted with this tumor (Figure 4, Panel B) and a further treatment with Na[*trans*-$RuCl_4$(DMSO)Im] of the same tumor line in intact hosts does not appreciably increase this effect (Figure 4, Panel C).

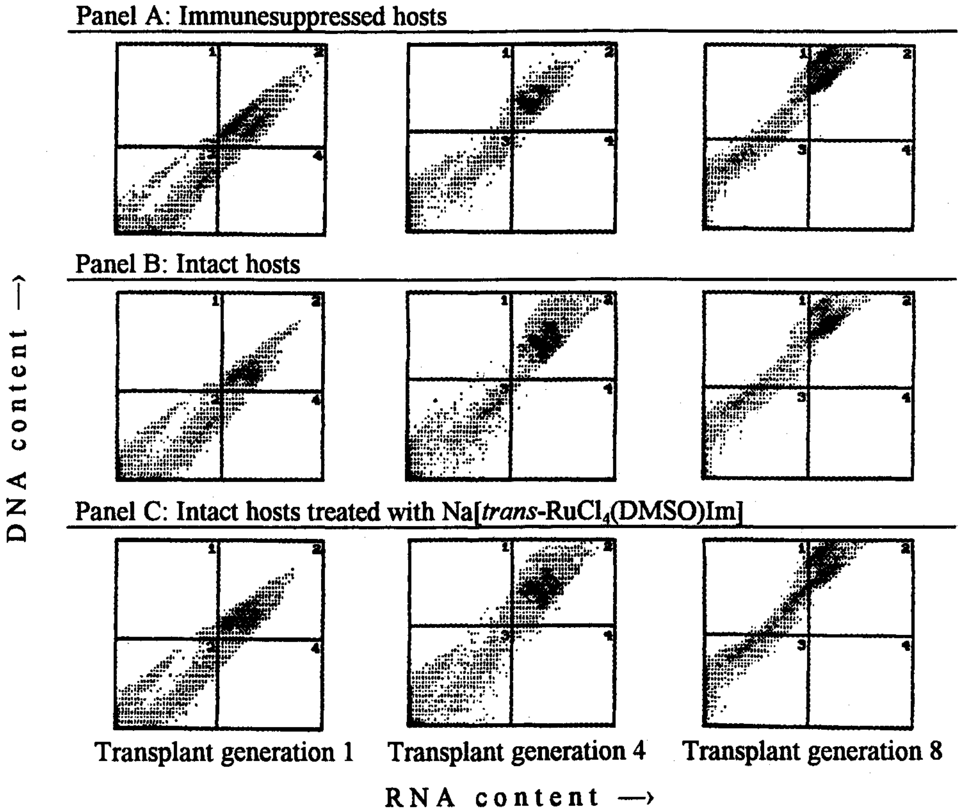

**Figure 4.** Effects of *in vivo* treatment of MCa mammary carcinoma with Na[*trans*-$RuCl_4$(DMSO)Im] for 8 transplant generations on DNA and RNA content. 3 mice of groups A and B of transplant generations of Figure 1 and of the group of mice immunesuppressed with DTIC were killed on day 14 and primary tumors removed, pooled and processed for the preparation of a tumor cell suspension stained with acridine orange and read with the cytofluorimeter.

Histological analysis. Sections of primary tumor of MCa mammary carcinoma obtained from DTIC immunosuppressed mice and treated with Na[*trans*-$RuCl_4$(DMSO)Im] were stained with Hematoxylin-Eosin and Cajal-Callego dyes. From the combined examination of the two stainings it appears that tumor growth progressively invades muscular fibers which however maintain their histological integrity. No modification of tumor cell shape, cell nucleus and staining was detected between untreated and treated mice. Furtheremore, no apparent difference of infiltrating leucocytes is noted between untreated or treated tumors or with increasing transplant generations.

Effects on TLX5 lymphoma. TLX5 lymphoma cells, treated *in vitro* with $10^{-4}$M Na[*trans*-$RuCl_4$(DMSO)Im] for up to 13 transplant generations, do not show any reduction of tumor take and growth in both intact or immunosuppressed hosts (Figure 5, Panel A). Similarly, no significant modification of the capacity of TLX5 lymphoma cells to metastasize to the brain was detected by means of a *vivo-vivo* bioassay of whole brains harvested from the test animals and aseptically transplanted into syngenic CBA intact mice (Figure 5, Panel B).

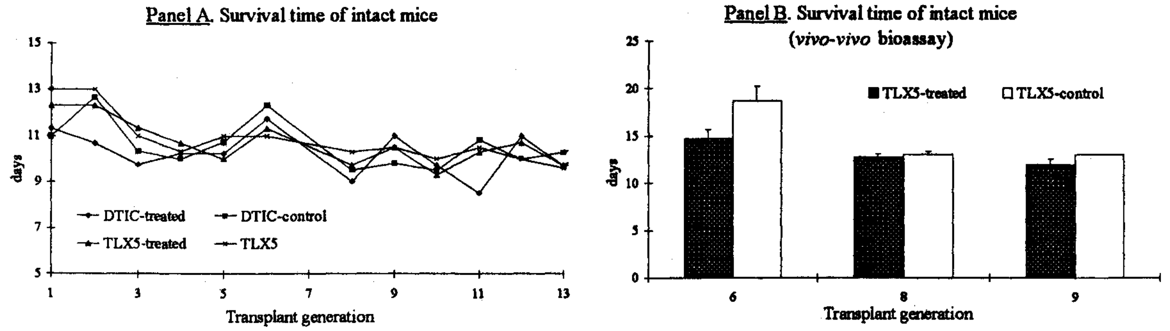

**Figure 5.** Effects of *in vitro* treatment of TLX5 lymphoma with Na[*trans*-$RuCl_4$(DMSO)Im] for 13 transplant generations on the survival time of mice *in vivo* transplanted with the treated tumor cells or with whole brains. Groups of 6 intact CBA mice or immunosuppressed with DTIC on day -2, transplanted i.p. with $10^5$ cells of TLX5 lymphoma previously challenged *in vitro* with $10^{-4}$ M Na[*trans*-$RuCl_4$(DMSO)Im], were examined for survival time (Panel A). 3 mice per group were killed on day 7 and their whole brains transplanted s.c. in intact hosts whose survival time is reported in Panel B.

### DISCUSSION

The examination of the effects of Na[*trans*-$RuCl_4$(DMSO)Im] on the growth of Mca mammary carcinoma showed a reproducible selective antimetastatic effect prevailing over the effect on the growth of primary tumors. No evidence was given yet as to stress the mechanism by which Na[*trans*-$RuCl_4$(DMSO)Im] blocks metastasis formation. Several possibilities may be put forward and one of them is the possibility that Na[*trans*-$RuCl_4$(DMSO)Im] modifies tumor cells, at primary site, causing the appearance of a tumor line with reduced metastasizing ability. A preliminary experiment seemed to suggest such possibility, showing that the transplantation of tumor cells obtained by *in vivo* treated mice into intact syngenic recipients gave rise to a normal growth of primary tumor but to a pronounced reduction of lung metastasis formation.

Therefore the present study was focused at evaluating the effects of the treatment for several transplant generations of the same tumor line on tumor growth and metastasis formation. TLX5 lymphoma, already shown to be a tumor line poorly responding to Na[*trans*-$RuCl_4$(DMSO)Im], was used because it offers good possibilities to highlight the occurrence of a chemical xenogenization similar to that described by the group of Fioretti (8,9). Despite 13 transplant generations in which TLX5 lymphoma cells were always exposed to $10^{-4}$M Na[*trans*-$RuCl_4$(DMSO)Im] for 1 hr no change of tumor growth and capacity to colonise the brain was detected. These data support the hypotesis that Na[*trans*-$RuCl_4$(DMSO)Im] does not induce new antigenicity to tumor cells and that lung metastasis formation of solid tumors are probably reduced by a mechanism different from xenogenization. In fact, data from a similar experiment performed with the solid MCa mammary carcinoma shows again that no apparent modification of tumor growth and of metastasis formation occur following *in vivo* treatment of tumor cells for up to 8 transplant generations. In the same model, however, it appears that the formation of spontaneous lung metastases becomes markedly more susceptible to the antimetastatic action of Na[*trans*-$RuCl_4$(DMSO)Im] after 4 transplant generations, as results by the examination of the response of the xenogenised tumor line to a further treatment with Na[*trans*-$RuCl_4$(DMSO)Im] in intact hosts. At the same time, it appears that the activity of DNA, but not that of RNA, in tumor cells markedly increased with the progression of the transplant generations.

Taken together these observations stress the already reported lack of cytotoxicity of Na[*trans*-$RuCl_4$(DMSO)Im] for tumor cells and suggest that the interaction of this compound with tumor cells does not induce resistence to the antimetastastic effect. Conversely it seems that the selection of a cell population with higher sensitivity to its antimetastastic action is unrelated to an appreciable modification of tumor take and growth in the syngenic hosts.

**ACKNOWLEDGEMENTS**

This work was done with contributions by MURST 60%, by CNR (contract 95.01689.CT04), MURST 40% (Metodi Innovativi in Farmacologia) and by Cassa di Risparmio di Trieste-Fondazione.

**REFERENCES**

1) G. Sava, S. Pacor, G. Mestroni and E. Alessio. *Clin. & Exp. Met.,* **10** pp. 273-280 (1992).
2) G. Sava, S. Pacor, G. Mestroni and E. Alessio. *Anticancer Drugs* **3** pp. 25-31 (1992).
3) G. Sava, S. Pacor, M. Coluccia, M. Mariggiò, M. Cocchietto, E. Alessio and G. Mestroni. *Drug Invest.* **8** pp. 150-161 (1994).
4) R. Gagliardi, G. Sava, S. Pacor, G. Mestroni and E. Alessio. *Clin. & Exp. Met.* **12** pp. 93-100 (1994).
5) G. Sava, S. Pacor, E. Alessio, G. Mestroni, R. Gagliardi, M. Cocchietto and M. Coluccia. *Drugs of the Future* **18** pp. 894-900 (1993).
6) J.E. Talmadge. *Cancer Met. Rev.* **2** pp. 25-40 (1983).
7) G.H. Heppner and B.E. Miller. *Cancer Met. Rev.* **2** pp. 5-24, (1983).
8) P. Puccetti, L. Romani and M.C. Fioretti. *Cancer Met. Rev.* **6** pp. 93-111 (1987).
9) P. Puccetti, L. Romani and and M.C. Fioretti. *Trends in Pharm. Sc.* **6** pp. 213-217 (1985).
10) E. Alessio, G. Balducci, M. Calligaris, G. Costa, W.M. Attia and G. Mestroni. *Inorganic Chem.* **30** pp. 609-618 (1991).
11) M. Poliak-Blazi, M. Boranic, B. Marzan and M. Radacic. *Vet. Arh.* **51** pp. 99-107 (1981).
12) M. Coluccia, G. Sava, F. Loseto, A. Nassi, A. Boccarelli, D. Giordano, E. Alessio and G. Mestroni. *Eur. J. Cancer* **29A** pp. 1873-1879 (1993).
13) G. Sava, T. Giraldi, L. Lassiani and C. Nisi. *Cancer Treat. Rep.* **66** 1751-1755 (1982).
14) A. Krishan. In: *Flow cytometry* (Z. Darzynkiewicz and H.A. Crissman. Eds), pp 121-125, Academic Press, San Diego (1990).
15) Z. Darzynkiewicz. In: *Flow cytometry* (Darzynkiewicz Z. and Crissman H.A. Eds), pp. 285-299, Academic Press, San Diego, 1990.

**Received: November 16, 1995 - Accepted: December 18, 1995 - Received in revised camera-ready format: January 30, 1996**

